# Introductory Lecture Delivered

**Published:** 1846-01

**Authors:** 


*Introductory Lecture delivered by* GUNNING S. BEDFORD, A. M., M. D., Professor of Midwifery, &c., in the New York University; session 1845–46. (From the author.)

This introductory of Dr. Bedford was published by request of the class, and is a strong appeal to student and practitioner in behalf of the department of science whose chair he fills. The Dr. strenuously denies that the analogy of the parturition of animals and the human female is sufficient to warrant the conclusion that the latter does not require medical aid, and establishes his position by well drawn arguments from the comparison of anatomical structure. Cases are cited in a style so powerfully graphic, and principles so clearly stated, as to impress indelibly upon his hearers or readers the necessity of a full acquaintance with all parts of Obstetrical science. We have room but for one quotation, though there is much of the lecture we would like to transfer to our pages. ED.

"Remember, however, that the duties of the accoucheur do not terminate with the delivery of the child; and fortunate would it be for the parturient woman if this doctrine were more generally inculcated. The opinion that the perils of the lying-in chamber cease with the birth of the fœtus is not only preposterous, but is fraught with danger both to the practitioner and patient. The management of the placenta constitutes, in itself, one of the nicest and most interesting points connected with the whole practice of midwifery. Tell me not that the delivery of the child emancipates the woman from all further peril. Truly has it been remarked, by a most emphatic and lucid author, that no man should have the hardihood to cross the threshold of the lying-in room who is not prepared, promptly and effectively, to conduct every placenta case that may by any possibility present itself. I respond most heartily, with all consciousness of its truth, to the value of its sentiment; and I would say to those, who have never yet been engaged in the practice of the profession, that if there be any one thing more than another, in the whole routine of professional duty, calculated to strike terror into the heart of the practitioner, and for a moment paralyse his best energies, it is a case of *flooding* after the birth of the child. Here there is no time for consultation—no time for reference to authority—one moment's hesitation or doubt on the part of the practitioner, and death speedily terminates the scene. Nature has opened her *flood-gates*, and if they be not instantly and skilfully closed, all chance of rescue is at an end. * * * The management of the after-birth not only involves the immediate safety of the female, but it frequently entails much future suffering and disease. A common, and occasionally a very formidable malady incident to the female sex—I mean *falling of the womb*—is, I am sure, in many instances, directly traceable to the indiscreet and hurried efforts of the accoucheur to extract the placental mass. The child is delivered; and, not content to wait the proper time for the uterus to throw off the after-birth, the medical attendant proceeds to remove it by forcible tractions. If these tractions do not result in hemorrhage, they often induce malposition of the womb, thus giving rise to a multitude of painful annoyances, which often terminate in serious, if not fatal, derangement of the uterine functions. I shall dwell, during the session, with emphasis on this subject, and shall call your attention, in an especial manner, to the rule of conduct to be pursued in all cases of difficulty, connected with the delivery of the placenta."

